# Life satisfaction, psychological stress, and present-moment attention: a generalizability study

**DOI:** 10.3389/fpsyg.2024.1258896

**Published:** 2024-02-19

**Authors:** Armin Jentsch, Frances Hoferichter

**Affiliations:** ^1^Department of Teacher Education and School Research, University of Oslo, Oslo, Norway; ^2^Department of Education, University of Greifswald, Greifswald, Germany

**Keywords:** life satisfaction, psychological stress, present-moment attention, generalizability theory, pre-service teachers

## Abstract

During the last decade, many teachers have retired early, leading to increased discussions about how to improve and maintain their mental health. To address this concern early, we designed an online seminar covering the field of positive psychology by emphasizing on mindfulness, positive emotions about one's future, and resources for pre-service teachers. The seminar was designed to increase their wellbeing, as well as to decrease psychological stress. To explore the sensitivity of our measures to change, we investigate the following research questions: To what extent do we assess trait or state variability in students' outcomes and what are the levels of reliability achieved? Fifty-four students in their second year at a German university (median age 22 years) participated and they were asked to fill in questionnaires assessing their life satisfaction, psychological stress, and present-moment attention during each of nine sessions over the course of a semester. We performed Generalizability and Decision Studies to estimate variability between-students and within-students, error of measurement, as well as reliability. Our results show that life satisfaction and psychological stress reached high reliability, suggesting that students' trait and state variability were both assessed with high accuracy. On the other hand, the assessment of present-moment attention would benefit from adding more items to the questionnaires or collecting data from more sessions. We discuss how our findings impact research and practice.

## 1 Introduction

Concerns regarding the health and wellbeing of teachers have grown in the past decade due to early retirement and permanent exits from the profession, resulting in a shortage of teachers and posing a threat to the quality of the education system. Teachers, compared to other professional groups, consistently report lower levels of psychological wellbeing (Johnson et al., 2005), higher work-related stress (Smith et al., 2000; Unterbrink et al., 2007), burnout and psychosomatic health complaints (Schaufeli, 2003). These impairments, in addition to structural and organizational factors within schools, greatly affect teacher retention and turnover. Since 2009, the number of teachers remaining in the profession at the beginning of their careers has steadily declined, with each new cohort experiencing a higher attrition rate than the previous one (Sims, 2018). Consequently, concerns about psychosomatic health are not limited to the early stages of a teaching career but also exist among pre-service teachers who are still studying to become educators.

To address this concern adequately, we designed a seminar that aims to strengthen pre-service teachers' life satisfaction, mindfulness, as well as reduce their psychological stress to increase their overall wellbeing. We assessed life satisfaction, present-moment attention as a facet of mindfulness, and psychological stress in students multiple times over the course of a semester to explore the generalizability of our measures (Cronbach et al., 1972), as well as the nature of the constructs being assessed.

### 1.1 Mindfulness, life satisfaction, and psychological stress

While life satisfaction refers to a person's overall evaluation and subjective perception of life, it is a subjective measure of wellbeing that encompasses a person's cognitive evaluation and emotional response to his or her life circumstances, achievements, relationships, and overall fulfillment (Proctor et al., 2017). Individuals who are satisfied with their lives are also more likely to cope effectively with stressors and therefore report lower levels of stress (Milas et al., 2021) as well as higher wellbeing (Ojha and Kumar, 2017).

Research such as a large-scale longitudinal study by Schaarschmidt (2004) has demonstrated that both pre-service and in-service teachers experience significant psychological stress and exhibit a notable lack of effective coping strategies compared to professionals in other fields. Likewise, Reichl et al. (2014) found that pre-service teachers possess less effective stress management techniques than those in other occupations, making them more susceptible to developing burnout syndromes throughout their teaching careers. Given the various stressors encountered by both pre-service and in-service teachers throughout their educational training and careers (Hoferichter, 2019; Wettstein et al., 2020; Jentsch et al., 2022), it becomes crucial to equip them with effective techniques and concepts that can be applied in typical settings of educational practice (Benevene et al., 2019).

Mindfulness refers to being fully present and engaged in the current moment, without judgment or attachment to thoughts, emotions, or sensations. It involves paying attention to one's experiences, both internal and external, with a sense of openness and acceptance (Seligman et al., 2005). Research investigating the link between mindfulness and wellbeing among students consistently finds a positive link (Rahe et al., 2022; Rehman et al., 2023).

In sum, life satisfaction, the ability to cope with stressors, and mindfulness present protective factors, especially in challenging situations, and support individuals in maintaining and strengthening their wellbeing.

### 1.2 Research questions

Developing measures that are generalizable across various assessment settings and better understanding the assessed construct are both important (e.g., trait vs. state variability), particularly if the measures are used for different purposes in research and practice (e.g., correlation studies, intervention, or individual assessment, see Kane, 2013).

We investigate the following three research questions for each of our three outcomes: (1) To what extent do we assess trait (i.e., variability between students) or state variability (i.e., variability within students) in students' outcomes? (2) What are the levels of reliability and dependability achieved, i.e., are the measures suited for relative or absolute inferences? (3) How many sessions or items are necessary to reach sufficient levels of reliability and dependability?

## 2 Method

### 2.1 Seminar outline

The seminar was a regular class for pre-service teachers in their second or third year at university. It involves course work on psychological stress (e.g., Lazarus and Folkman, 1984), positive psychology (Seligman et al., 2005), as well as case studies and individual reflections. The seminar focuses on topics like wellbeing, self-efficacy, and building resilience to equip pre-service teachers with coping strategies for the demands of their future jobs. The seminar is not a mandatory class but can be chosen deliberately by pre-service teachers out of a collection of several classes covering various topics in educational research (e.g., pedagogy, educational psychology, and research methodology). The seminar was held online albeit synchronous during nine sessions (~2 h each), and every session followed a similar agenda: (1) light exercises (e.g., yoga), (2) lecture (i.e., short input of the topic to be covered), (3) group discussion and/or reflection, (4) introduction and application of mindfulness-based and stress-reducing methods. In addition, pre-service teachers were assigned tasks for homework in between sessions to be documented in a diary.

### 2.2 Measures

#### 2.2.1 Life satisfaction

Life satisfaction was assessed with the General Life Satisfaction Short Scale (L-1, Beierlein et al., 2015). It comprises one item (“All things considered, how satisfied are you with your life these days?”) that is rated on an eleven-point scale ranging from “not at all satisfied” (0) through “completely satisfied” (10). A large body of research is available on that provides evidence on the validity of the L-1 for different purposes of assessment (e.g., Jang and Kim, 2009; Beierlein et al., 2015; Soto and John, 2017).

#### 2.2.2 Psychological stress

Psychological stress was assessed with the question “How stressed are you now?” which was taken from Ecological Momentary Assessment (EMA, e.g., Mennis et al., 2018) survey. The question is answered on a seven-point scale ranging from “not at all stressed out” (0) to “very stressed out” (6). Mennis et al. (2018) showed that the scale can efficiently be used to measure psychological stress in various population subgroups.

#### 2.2.3 Present-moment attention

Present-moment attention was assessed with the Multidimensional State Mindfulness Questionnaire (MSMQ, Blanke and Brose, 2016) after each seminar session. The original questionnaire comprises three dimensions and nine items, but we only used the three items measuring present-moment attention (item 1: “I focused my attention on the present moment”, item 2: “I concentrated on what I was doing at that moment”, and item 3: “I took note of my thoughts and feelings”). The items were answered on a nine-point scale ranging from “does not apply at all” (0) to “applies strongly” (8). Blanke and Brose (2016) argue that the MSMQ is particularly well suited to assess state mindfulness, as they found only low to moderate correlations with measures of trait mindfulness in a population of German university students.

### 2.3 Participants and procedure

The study was conducted in line with the principles of the Declaration of Helsinki. As this is an observational study, no additional ethical approval is required. Informed consent was obtained from all individual participants. Fifty-four pre-service teachers (38 females) from a university in North-Eastern Germany took part in our study. They were in their second or third year of a teaching program for secondary schools (32 students) and had a median age of 22 years. Questionnaires were administered online and within the learning platform where the seminar took place. Life satisfaction and psychological stress were assessed twice per session (pre and post), and present-moment attention was assessed once, after each session. Dropout in the post-session questionnaire was close to 40%. On average, the students attended six out of the nine sessions in our seminar (ranging from 1 through 9). Table 1 provides further sample characteristics.

**Table 1:** Sample characteristics (*n* = 54 pre-service teachers).

Sex female/male	38/16
Academic track low/high	22/32
**Age (years)**	
Median (range)	22 (18−49)
**Life satisfaction (0**−**10)**	
Pre *M* ±*SD*	5.22 ± 1.64
Post *M* ±*SD*	5.49 ± 1.56
**Psychological stress (0**−**6)**	
Pre *M* ±*SD*	3.62 ± 1.51
Post *M* ±*SD*	3.08 ± 1.35
**Present-moment attention (0**−**8)**	
Total score *M* ±*SD*	5.12 ± 1.88
Item 1 *M* ±*SD*	5.71 ± 1.70
Item 2 *M* ±*SD*	5.69 ± 1.66
Item 3 *M* ±*SD*	3.96 ± 1.72

### 2.4 Generalizability and decision studies

We conducted separate generalizability and Decision Studies (G and D Studies, Cronbach et al., 1972) using IBM SPSS 29 to address our research questions. For each of the three outcomes, we estimated random student and session effects (i.e., variance components). This resulted in a fully nested design for life satisfaction and psychological stress, which is pre/post assessments within sessions within students. For present-moment attention we estimated additional item main and interaction effects in a partially nested design. As suggested for small-scale studies with missing data (Brennan, 2001), ANOVA-type estimators were applied. The literature suggests using the resulting variance components to estimate error of measurement and reliability (or dependability, Cronbach et al., 1972). G Studies allow for the estimation of two types of error (namely, absolute, and relative, with the former being slightly more conservative, Brennan, 2001).^1^ Reliability is then defined as student variance divided by student variance plus relative error. Dependability is defined as student variance divided by student variance plus absolute error. By conducting exploratory D studies, we can investigate how many sessions or items (or combinations thereof) are necessary to achieve sufficient levels of reliability and dependability. In this study, values of 0.75 are considered acceptable.

## 3 Results

**Figure 1:**
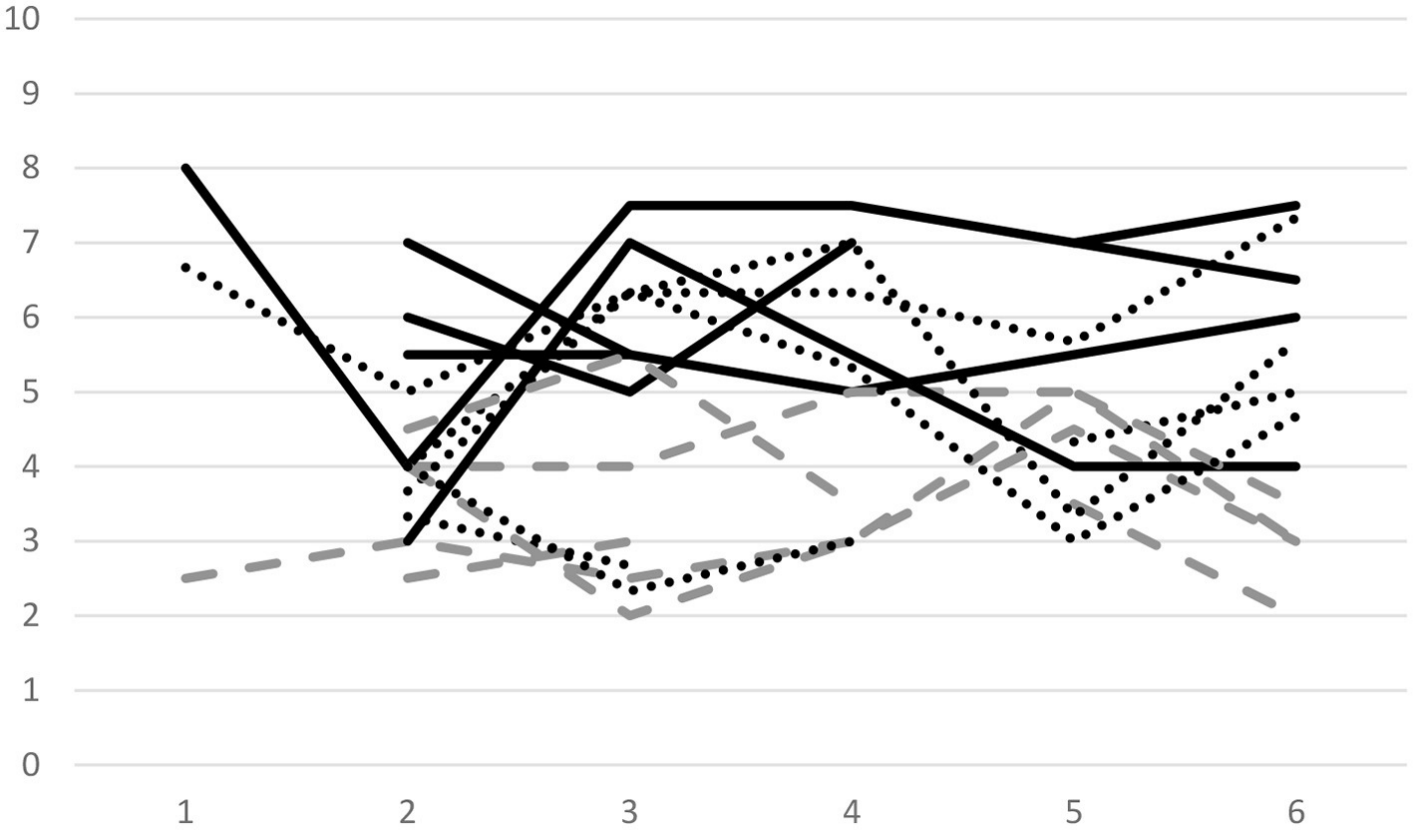
Individual profiles of life satisfaction (solid lines), psychological stress (broken lines), and present-moment attention (dotted lines) total scores per session for five randomly selected students and six sessions. Pre- and post-measures within sessions were averaged for life satisfaction and psychological stress.

Figure 1 shows five randomly selected individual profiles on all outcome variables over the course of six sessions. We see that there is a large amount of both between and within person variability across the three outcomes. However, at the same time our results indicate no clear time trend for any of the variables. G studies should provide further insights into the distribution of variance regarding students' traits and states.

Table 2 presents the findings from the G Studies. The estimated variance components explain between 60 and 80% of the variability in student outcomes. For life satisfaction, the variability between students and sessions (i.e., within students) each account for about 30% of the total variance. For psychological stress, between-session variability reaches similar values, but differences between students explain almost half of the total variance. This results in estimated reliability and dependability coefficients close to 0.80 for life satisfaction and psychological stress. We note again that although life satisfaction and psychological stress were measured twice in every session, within-session variance cannot be estimated, but is confounded with the residuals. Thus, differences within sessions could account for up to 20–40% of the total variability in student outcomes.

**Table 2:** G and D Studies for life satisfaction, psychological stress, as well as present-moment attention.

	**Life satisfaction**		**Psychological stress**		**Present-moment attention**	
	**Estimate**	**%**	**Estimate**	**%**	**Estimate**	**%**
Students	0.650	31.0	1.263	47.4	0.485	12.6
Sessions	0.587	28.0	0.833	31.3	0.727	18.9
Items	–	–	–	–	1.298	33.7
Students × items	–	–	–	–	0.337	8.7
Residuals	0.857	41.0	0.569	21.3	1.225	31.8
Total	2.094	100.0	2.665	100.0	3.857	100.0
Relative error	0.160		0.156		0.238	
Absolute error	0.160		0.156		0.671	
Reliability	0.780		0.794		0.670	
Dependability	0.780		0.794		0.419	

Reliability is student variance divided by student variance plus relative error. Dependability is student variance divided by student variance plus absolute error (see Section 2.4).

Present-moment attention provides a different picture in this study. Most of the variability in scores seems to be due to differences between items. A similar amount of variance remains unexplained. About 13% of the total variability could be attributed to differences between students, and 19% were due to variability across sessions (i.e., within students). However, <10% of the total variability were due to students applying item scales differently for present-moment attention. This suggests that the items within the scale assess various facets of present-moment attention that do not necessarily overlap. Altogether the findings point to the fact that our assessment of present-moment attention is suited to capture rankings of students to a limited extend (i.e., reliability of 0.67). However, the findings also show that the dependability of the assessment is low. This suggests that scores should not be used for absolute inferences (see Table 2).

**Figure 2:**
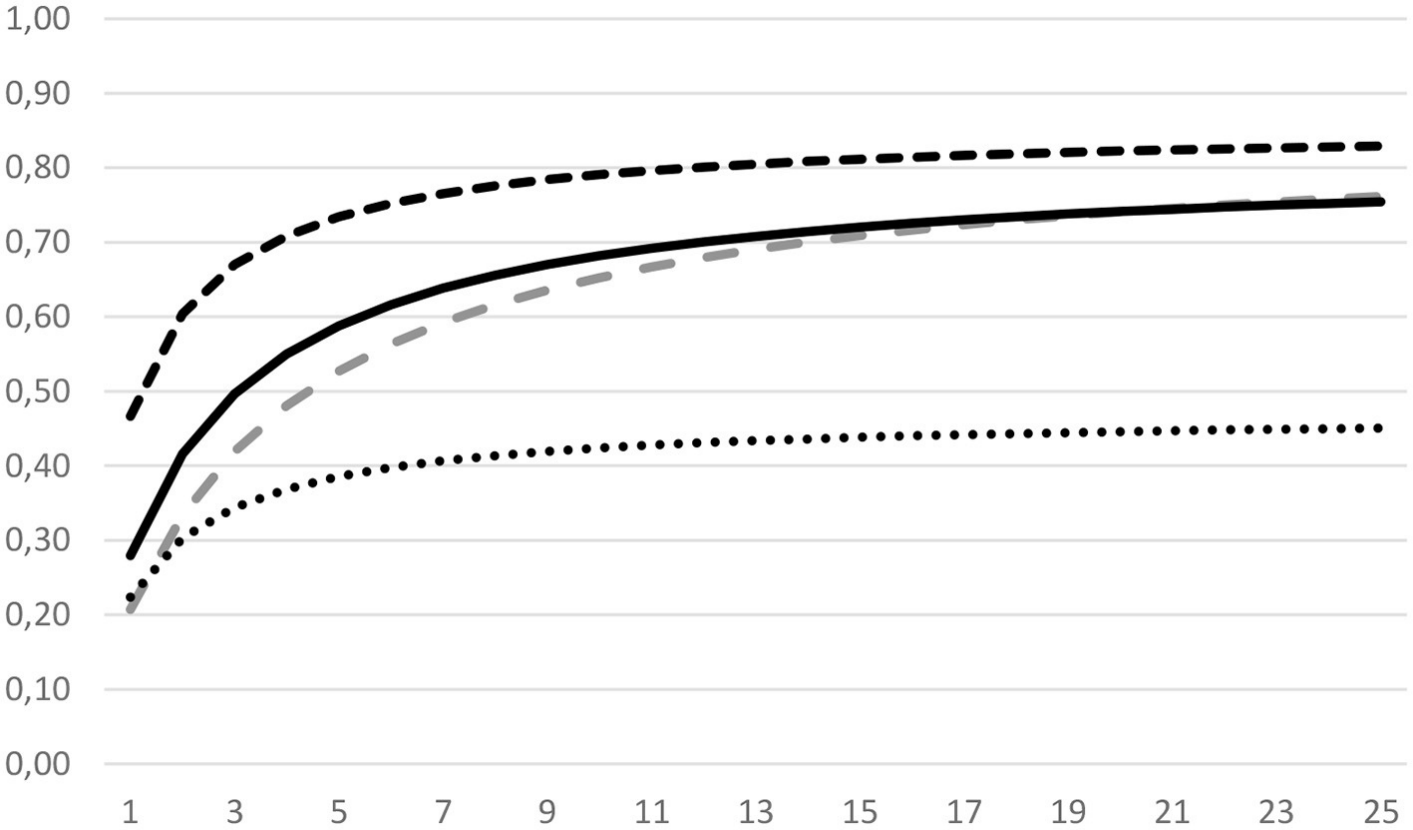
Reliability (upper two graphs) and dependability coefficients (lower two graphs) for present-moment attention with varying number of items (broken lines) or sessions (solid and dotted lines). Thus, the top graph represents the reliability for present-moment attention with an increasing number of items (fixed at nine sessions), and the bottom graph represents the dependability of present-moment attention with an increasing number of sessions (fixed at three items).

As pointed out in Section 2.4, we conducted additional exploratory D studies for all measures that did not result in sufficient reliability or dependability. Thus, D studies for present-moment attention were performed regarding the number of items and sessions which contributed to the total variance in the assessment. Figure 2 shows that by employing about 10 items, sufficient reliability could be achieved for present-moment attention (i.e., with a fixed number of sessions). Over the course of about 20 items or sessions, sufficient reliability and dependability could be reached for present-moment attention. However, the assessment would result in insufficient dependability, even with more than 20 sessions.

## 4 Discussion and limitations

Teacher wellbeing is an important topic in the field of education, as pre-service teachers already struggle often with mental health issues and stress (Schaarschmidt, 2004; Reichl et al., 2014). We collected intensive longitudinal data over the course of a semester to learn about students' perceptions of life satisfaction, stress, and present-moment attention (Beierlein et al., 2015; Blanke and Brose, 2016; Mennis et al., 2018), and to explore how sensitive our measures are to change. To decompose variability in student outcomes, we employed a G study framework that also allowed us to estimate reliability and dependability coefficients. We found little evidence for time trends in our study, which is particularly interesting regarding students' present-moment attention. Blanke and Brose (2016) developed this measure specifically to assess state variability and therefore, we would have expected to find a larger share of variation within students over the course of a semester.

One reason for this finding could be that many students did not attend the full course (i.e., missing data), which makes it difficult to draw conclusions on students' individual trajectories regarding the assessed constructs. As a result of the dropout, individual students might not have made the intended progress during the course, and therefore report similar levels of life satisfaction, stress, and present-moment attention over time. This suggests small shares of within-student variability in the respective constructs, as reported in this study. Further research is necessary to shed light on how students develop over the course of a semester or year regarding relevant outcomes.

Furthermore, another reason could be that the measures do in fact assess state variability, in the sense that students found themselves in similar “states” during different measurements. For instance, they could have been exposed to various stressful situations in the beginning (e.g., organizing their schedules), mid-term, and the end of the semester (e.g., exams). Thus, the data might reflect recurrent, but ever-changing “daily hassles” that affect students' self-reports on their satisfaction and stress. In our opinion, these “daily hassles” could be investigated by first employing additional, qualitative research methods (e.g., interviews, student diaries, see Hoferichter, 2019; Wettstein et al., 2020), and second, by exploring each of the reported sources of psychological stress in more detail.

Another reason could be that although state measures were applied, we largely captured variability in students' traits, which is suggested by the results presented in Table 2. While this is potentially true for students' perceptions of satisfaction and stress, it might be unlikely that students' (trait) mindfulness changed over a short time. Mindfulness was just one topic out of seven in our seminar, and it needs a large amount of motivation and personal effort to deliberately practice mindfulness such that it becomes a daily routine. However, it could be interesting to follow up on this finding by either investigating other facets of mindfulness (e.g., in terms of their sensitivity to change, which is likely to depend on the facet), employing or developing additional measures of present-moment attention, or by replicating our results with samples from other populations. Mindfulness has been shown to be an important protective factor for wellbeing and life satisfaction after all (Rahe et al., 2022; Rehman et al., 2023).

It is important to also consider several ramifications of this study. First, the generalizability of our findings is limited. As the sample consisted of pre-service teachers from a single university, more research is needed to better understand to what extent the reported results depend on the study setting. Future studies could investigate whether the measures used in this study are also suitable to assess life satisfaction, psychological stress, and present-moment attention in schoolteachers.

Second, the self-selection of the study participants could have biased our findings in two ways. On one hand, students might have been more motivated to work on their mental health because they volunteered to take part in our study. On the other hand, they were of course aware of participating in a study on wellbeing and therefore, social desirability might have influenced their responses (Hawthorne effect). However, we note again that our aim was not to test for an intervention effect but to discuss the sensitivity of the measures to change, for which we found mixed results in the present study.

Third, mindfulness is considered a multi-faceted and complex construct, and we assessed only one facet in our study (i.e., present-moment attention). The results suggests that it needs more items to measure this facet with sufficient dependability. An alternative to adding more items to the scale would be to treat item variability as fixed effects. G Theorists usually suggest doing so if the variability of a facet is considered large (Cronbach et al., 1972; Brennan, 2001). A solution to the issue of low reliability under this assumption is to either estimate separate means and variance components for each item, or to average across them. In the present study, however, we have estimated item random effects to discuss their contribution to measurement error.

## Data availability statement

The raw data supporting the conclusions of this article will be made available by the authors, without undue reservation.

## Ethics statement

Ethical approval was not required for the studies involving humans because this research involves the use of non-sensitive, anonymous surveys, and participants not defined as vulnerable. Their study participation was considered not likely to induce undue psychological stress or anxiety. The studies were conducted in accordance with the local legislation and institutional requirements. The participants provided their written informed consent to participate in this study.

## Author contributions

AJ: Data curation, Formal analysis, Methodology, Writing – original draft. FH: Funding acquisition, Project administration, Writing – review & editing.

## Footnotes

^1^It should be noted that in this study, absolute and relative error is the same for life satisfaction and psychological stress due to a fully nested design. This also suggests that estimating reliability and dependability coefficients results in the same values.
